# Accidental Employment of Chloroform in Erysipelas

**Published:** 1851-05

**Authors:** Geo. J. Sacshe

**Affiliations:** Columbus, O.


ARTICLE IV.

ACCIDENTAL EMPLOYMENT OF CHLOROFORM IN ERYSIPELAS.—By GEO. J. SACSHE. M. D., Columbus, O. Translated from the German by JOHN KERN.

On the 31st December, 1850, I was called to visit Mrs. K., aged about 45, who had been laboring under vesicular Erysipelas of the face for three days. There was considerable febrile excitement, constipation, tongue brown and dry, and skin having a yellowish tinge.

Prescribed Cal. gr. xii., followed by an infusion of Sal Epsom and Senna. After free action of the bowels, she was placed under gentle diaphoretics, and acidulated drinks. The disease spread somewhat upon the face, but by the influence of this treatment, at the end of ten days my patient was convalescent.

On the 25th of January, I was again summoned to see Mrs. K., who, the day before, had had another attack of Erysipelas upon the left side of the face. The inflamed surface was covered by a fine vesicular eruption. I administered an emetic of Tartar and Ipecac., which produced free vomiting and catharsis. In the afternoon the disease was found to be spreading—the face was much swollen. I resolved at once to use the colodion, which I had brought with me for that purpose. I removed the cork from what I supposed to be my colodion bottle, and by dipping the extremity of a feather into the fluid, I applied it to the inflamed part, but to my surprise, it did not form a dry, shining, transparent pellicle, or coating, upon the surface—but in place of it, a whitish sediment, formed similar to that observed after the application of Goulard's extract. The redness, however began rapidly to subside. For the first time, my olfactories detected my mistake—I was using chloroform instead of colodion. This startled me not a little, but observing the happy effect of my new remedy, I continued its application.

On the following morning I found my patient much better. The inflammation had nearly subsided, except at certain points, to which she desired me to re-apply this "blessed water." On the third morning all redness had entirely disappeared.

Five days after this, I had another case of Erysipelas of the face in the person of a woman of 32 years of age. The redness of the inflamed surface was not as deep as in the first case. The chloroform was applied more freely, but twice only, on two consecutive days. The effect was quite as salutary as in the case of Mrs. K.

I would not propose the external application of chloroform as a sovereign remedy in Erysipelas, by any means. Neither am I certain that it would be generally useful. My experience thus far in its employment would not lead to such extravagant conclusions. Its happy effect in the above instances, or the favorable change which followed its use, encourage me to repeat its application in cases which may hereafter fall under my observation; and also induces me, in this manner, to call the attention of the profession to it. The suggestion that the history of this accidental employment of chloroform should be published, was made to me by a highly esteemed professional friend, whose wishes I feel bound to respect. If this article proves equally as efficacious as nitrate of silver, or colodion; on account of unpleasant local effects which these produce, it would be preferable to them, as it leaves no discoloration or tenacious coating on the part.

It is not to be expected that any local agent, however salutary, will obviate the necessity for constitutional remedies. In cases where there are disordered digestion, bilious and vascular derangements, and excitement, internal remedies appropriate to the existing indications, must not be omitted.—*Ohio Med. and Surg. Journal, March*, 1851.

